# The effect of conditional cash transfers on tuberculosis incidence and mortality is determined by ethnoracial and socioeconomic factors: a cohort study of 54 million individuals in Brazil

**DOI:** 10.21203/rs.3.rs-4272509/v1

**Published:** 2024-04-26

**Authors:** Davide Rasella, Gabriela Jesus, Priscila Pinto, Andréa Silva, Daniella Cavalcanti, Iracema Lua, Maria Ichihara, Mauricio Barreto, Delia Boccia, Mauro Sanchez

**Affiliations:** ISGlobal; Faculty of Medicine, Federal University of Bahia (UFBA); Institute of Collective Health, Federal University of Bahia (ISC/UFBA); Institute of Collective Health, Federal University of Bahia; Center for Data and Knowledge Integration for Health (CIDACS), Gonçalo Moniz Institute, Oswaldo Cruz Foundation (FIOCRUZ), Salvado; Institute of Collective Health, Federal University of Bahia (ISC/UFBA); Institute of Collective Health, Federal University of Bahia; Center for Data and Knowledge Integration for Health (CIDACS), Gonçalo Moniz Institute, Oswaldo Cruz Foundation (FIOCRUZ); The Center for Data and Knowledge Integration for Health (CIDACS), Oswaldo Cruz Foundation (FIOCRUZ); Center for Data and Knowledge Integration for Health (CIDACS), Gonçalo Moniz Institute, Oswaldo Cruz Foundation (FIOCRUZ); Faculty of Population and Health Policy at London School of Hygiene and Tropical Medicine (LSHTM); University of Brasília

**Keywords:** Tuberculosis, Conditional Cash Transfer, Bolsa Família Program, Social Determinants of Health, Brazil

## Abstract

**Background.:**

Conditional Cash Transfers (CCT) are the world’s most widely implemented interventions for poverty alleviation. Still, there is no solid evidence of the CCT effects on the reduction of the burden of Tuberculosis (TB) in marginalized and extremely vulnerable populations. We estimated the effect of the *Bolsa Família Program* (BFP), the largest CCT in the world, on TB incidence, mortality, and case-fatality rate using a nationwide cohort of 54.5 million individuals during a 12-year period in Brazil.

**Methods.:**

We selected low-income individuals who entered in the 100 Million Brazilians Cohort and were linked to nationwide TB registries between 2004 to 2015, and compared BFP beneficiaries and non-beneficiaries using a quasi-experimental impact evaluation design. We employed inverse probability of treatment weighting (IPTW) multivariable Poisson regressions, adjusted for all relevant socioeconomic, demographic, and healthcare confounding variables - at individual and municipal level. Subsequently, we evaluated BFP effects for different subpopulations according to ethnoracial factors, wealth levels, sex, and age. We also performed several sensitivity and triangulation analyses to verify the robustness of the estimates.

**Findings.:**

Exposure to BFP was associated with a large reduction in TB incidence in the low-income individuals under study (adjusted rate ratio [aRR]:0.59;95%CI:0.58–0.60) and mortality (aRR:0.69;95%CI:0.65–0.73). The strongest BFP effect was observed in Indigenous people both for TB incidence (aRR:0.37;95%CI:0.32–0.42), and mortality-aRR:0.35;95%CI:0.20–0.62), and in Black and Pardo people (Incidence-aRR:0.58;95%CI:0.57–0.59; Mortality -aRR:0.69;95%CI:0,64–0,73). BFP effects showed a clear gradient according to wealth levels and were considerably stronger among the extremely poor individuals for TB incidence (aRR:0.49, 95%CI:0.49–0.50) and mortality (aRR:0.60;95%CI:0.55–0.65). The BFP effects on case-fatality rates were also positive, however without statistical significance.

**Interpretation.:**

CCT can strongly reduce TB incidence and mortality in extremely poor, Indigenous, Black and Pardo populations, and could significantly contribute to achieving the End TB Strategy targets and the TB-related Sustainable Development Goals.

## Introduction

The COVID-19 pandemic was one of the main drivers of the global increase in extreme poverty and social disparities with devastating effects on Low- and Middle-Income countries (LMICs).^1^ Recovery from this crisis has been hampered by high food and energy prices as a result, in part, of the new conflicts and by the climate change effects in countries that are major food producers.^2^ We are currently living in what is defined as “the polycrisis era”, characterised by multiple crises interconnecting and synergizing between them at the global level. Tackling this polycrisis requires greater efforts and investments in social policies to protect the poorest populations, in particular in programs such as conditional cash transfers (CCTs).^2^

The health system disruption caused by the COVID-19 pandemic negatively impacted tuberculosis (TB) prevention and care.^1^ For the first time in 2020, the World Health Organization (WHO) announced an upward trend in TB incidence and mortality and a decline in the reported number of people newly diagnosed with TB,^3^ a phenomenon that was particularly intense in LMICs^1^ and also occurred in Brazil.^4^ This situation will increase the number of people with undiagnosed and untreated TB, leading to increased transmission of the disease and its severity.^3^ In particular, the COVID-19 pandemic has raised attention on how deteriorating social determinants of health (e.g., education, poverty, access to healthcare) could have influenced both TB incidence and mortality and has shown the importance of social protection when responding to global health challenges.^3^ Social protection, poverty alleviation, and multisectoral actions on broader TB determinants are acknowledged as key pillars of the End TB strategy by 2035, i.e., they are essential to reduce the TB burden.^3^

Since 2004, Brazil has implemented one of the world’s largest CCTs programs, the *Bolsa Família Program* (BFP). The primary goal of the BFP is to alleviate poverty by providing cash transfers along with requirements related to education and healthcare. The governmental program provides direct cash transfers to poor households with income below the poverty line defined by the Brazilian government as families earning between United States (US) dollars $18–36 per person per month (at an exchange rate of 5 Brazilian Real to 1 US dollar). The monthly cash benefits vary from US$17 to a maximum of US$41, depending on the size and composition of the household. In order to continue receiving the benefits, BFP beneficiaries have to fulfil specific conditionalities related to the healthcare for pregnant women (carrying out prenatal care) and children (compliance with the national vaccination schedule, monitoring nutritional status), and education (school attendance) for children and adolescents (Appendix p.3).^5,6^ The BFP has been able to improve the wellbeing of families in poverty, and to reduce social and income inequalities in society, improving access to education, food, and health services.^6^ Several studies have demonstrated the positive effects of BFP on health outcomes such as child mortality^7^, cardiovascular diseases,^8^ suicide,^9^ leprosy^10^ and some aspects of the TB burden^11–13^ among others. However, the effects of CCT on TB outcomes among populations at high risk for TB and with limited access to health services, such as marginalised groups and extremely poor populations,^14^ have never been systematically estimated.

This study aimed to evaluate the comprehensive effect of the BFP, one of the largest CCT programs in the world, on Tuberculosis incidence, mortality, and case-fatality rates using an unprecedented cohort of 54.5 million low-income Brazilians over 12 years, estimating its heterogeneous effectiveness across the spectrum of ethnoracial factors and socioeconomic conditions.

## Methods

### Study design, population, and ethical issues

**Figure 1.**
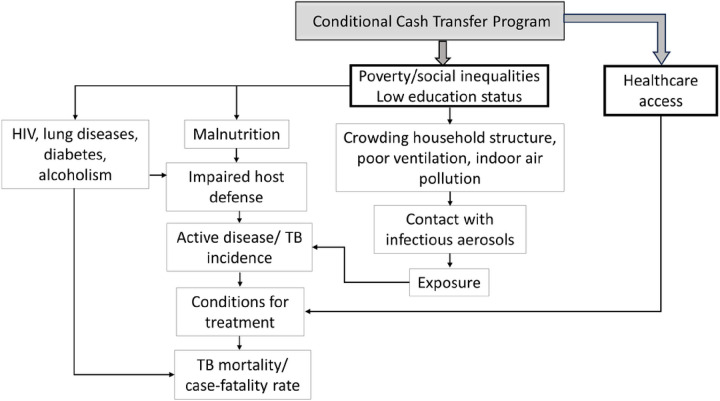
Conceptual framework about determinants and possible effects of the conditional cash transfers (CCT) on tuberculosis (TB) outcomes

This study has a quasi-experimental cohort study design, based on the longitudinal information of 54.5 million individuals from January 1, 2004 to December 31, 2015 (the period for which tuberculosis data were available). First, we constructed a conceptual framework to explain the mechanisms of possible effects of CCT on TB outcomes and to drive the analysis (Figure 1).^15^ The study population was achieved by selecting a subgroup of individuals of the 100 Million Brazilians Cohort^16^, a consolidated cohort created through the validated linkage^17^ between the Federal Government Unified Registry for Social Programs (*Cadastro Único*) – that gathers data from the poorest half of the Brazilian population, identifying and characterising low-income families for social programs eligibility, and including information on exposure to the BFP - and health-related datasets from the Brazilian Ministry of Health’s (Appendix, p.3).

This study was approved by the Research Ethics Committee of the Institute of Collective Health of the Federal University of Bahia (ISC/UFBA), under number 41691315.0.0000.5030 (Assessment nº:3.783.920).

### Data sources, outcomes, and intervention

Two individual-level health-related datasets were linked to *Cadastro Único* (CADU): the Notifiable Diseases Information System (SINAN) and the Mortality Information System (SIM). SINAN contains records of notifiable diseases, including TB. SIM registers deaths by all causes, according to International Classification of Diseases (ICD-10). The linkage codes and algorithms were built based on five identifiers: date of birth, municipality of residence, sex, name, and mother`s name of the individual in each database. The CADU and the health information datasets (SIM and SINAN) were individually matched in two steps, using the CIDACS-Record Linkage tool (Appendix, p.3). The quality of each link between CADU, SINAN, and SIM has been extensively evaluated and validated.^17^ An aggregate-level longitudinal dataset - containing a wide range of yearly municipal-level information on TB endemicity levels, municipal infrastructures, and healthcare resources - was also linked to the cohort through the individuals` municipal code of residence.

Tuberculosis outcomes defined for the study were: incidence, mortality, and case-fatality rates. The beneficiary group was defined as eligible individuals who received BFP benefits, and their exposure started with receipt of the benefit, until the end of their follow-up. The non-beneficiary group was defined as individuals who had never benefited from BFP throughout their follow-up period. In case of non-receipt of the benefits, eligible individuals were classified in the non-beneficiary group (Appendix p.4).

### Statistical Analyses

First, in the descriptive analysis, we estimated the rates of the study outcomes as follows: i) TB incidence: new TB diagnoses divided by person-years at risk and multiplied by 100,000; ii) TB mortality: TB deaths, divided by person-years at risk and multiplied by 100,000; and iii) case-fatality rate: TB deaths among people affected by TB, divided by person-years at risk and multiplied by 100. The follow-up time for each individual in the cohort, i.e., person-years, started on the date of entry into the cohort until the date of TB diagnosis (for TB incidence), the date of death due to TB (for TB mortality rate), the date of death from other causes, or the end date of the cohort (December 31, 2015). For TB case-fatality rate, the start date began with the date of diagnosis and ended with the TB-related death, the date of death from other causes, or the final date of the cohort. Afterwards, we performed a descriptive analysis of new people affected by TB and deaths according to each independent variable. At the individual level, the demographic and socioeconomic covariables were age, sex, self-identified race/ethnicity (white, Indigenous, Black and pardo - these last categories were analysed together), education, *per capita* expenditure (as a proxy for the *per capita* wealth and calculated as a percentage of the yearly minimum wage, categorised by tertiles), and year of entry into the cohort. At the family level, the independent variables were related to household characteristics: number of people, water supply, construction material, sanitation, garbage disposal, and lighting. At the municipal-level, the covariables were unemployment rate, Gini Index, and a set of variables related to health services: Family Health Strategy coverage (the main model of Primary Health Care in Brazil), number of doctors, nurses, and specialised clinics per 1,000 inhabitants. To control for any potential selection bias associating PBF implementation with endemic TB levels in the community, the mean TB incidence rate in the cohort during the study period was included as a covariate in the models. When the study outcome was the case-fatality rate, we also included clinical classification of TB, percent of directly observed therapy (DOT), AIDS comorbidity, and diabetes as independent variables. All the variables used in the study are described in the conceptual model (Figure 1).

To estimate the effect of BFP exposure on TB incidence, mortality, and case-fatality rates we used multivariable Poisson regression models, adjusted for all the relevant demographic and socioeconomic confounding variables listed above, with follow-up time as an offset variable, robust standard errors, and observations weighted through stabilised, truncated, inverse probability of treatment weighting (IPTW). Poisson regression models are common for cohort data analyses,^18^ and IPTW Poisson regression models have been used in quasi-experimental cohort studies which investigate the impacts of public and social policies on health outcomes, including several evaluation studies that used the 100 Million Brazilian Cohort.^8–10,19^ Moreover, in order to understand BFP effects heterogeneity, we fitted these IPTW Poisson regression models stratified by age, sex, race/ethnicity, education, and wealth- tertiles (*per capita* expenditure).

To confirm the robustness of the findings, we applied several sensitivity analyses (for details see Appendix, p.9–12): i) we fitted models with only individual-level variables and tested the inclusion of different aggregate-level variables, ii) we fitted the same regressions without the TB endemicity level variable, iii) we estimated and compared all models without IPTW, iv) to evaluate the adoption of *per capita* expenses as a proxy for wealth, we carried out the same analyses with other proxies, such as *per capita* income, v) we adjusted the same models with different specifications (including different sets of individual-level covariates, inclusion or exclusion of robust standard errors, only in municipalities with adequate vital information). Finally, to have a greater degree of confidence in the causal inference of our impact evaluation, we performed two different triangulation analyses,^20^ verifying the existence of BFP effects also using alternative methods: survival analysis with Cox multivariate regression and propensity score matching (PSM) (Appendix, p.13–14).

### Role of the funding source

The funding source had no role in study design, data collection, data analysis, data interpretation, or the writing of the report.

## Results

**FIGURE 2.**
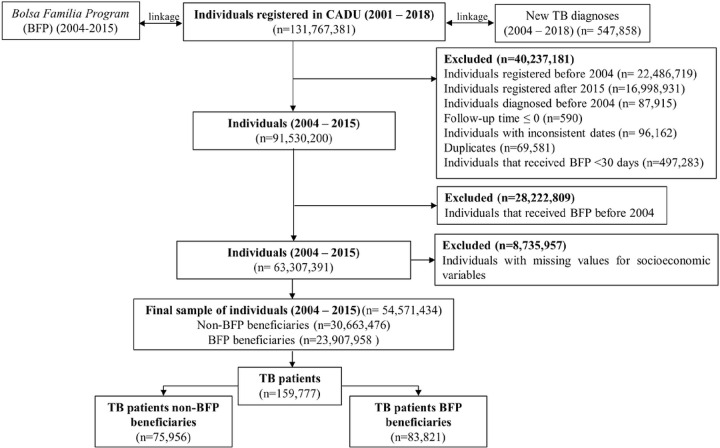
Flowchart selection of the study population, The 100 Million Brazilians Cohort, 2004–215

After excluding individuals of the 100 Million Brazilians Cohort who were outside the study period 2004–2015, and who had missing information on demographic or socioeconomic variables, 54,571,434 individuals were selected, of which 23,907,958 were BFP beneficiaries (43.8%), and 30,663,476 non-BFP beneficiaries (56.2%), with a total of 159,777 new TB diagnoses and 7,993 TB deaths (Figure 2). BFP beneficiaries and non-beneficiaries showed similar demographic and socioeconomic characteristics (Table 1). BFP beneficiaries had a slightly higher percentage of people self-identified as Black or pardo race/ethnicity, people with no education, households with 3 or more individuals, lesser wealth, without adequate sanitation, and without a public network for water supply (Table 1). TB incidence was lower among BFP beneficiaries than BFP non-beneficiaries (59.07/100,000 person-years at risk [pyr] vs 34.32/100,000 pyr), as well as TB mortality rate (3.99/100,000 person-years at risk [pyr] vs 1.89/100,000 pyr), while TB case-fatality rate was higher among non-beneficiaries (0.68/100,000 pyr vs 1.37/100,000 pyr).

In the IPTW multivariable Poisson regression models (Table 2), exposure to BFP was associated with a significant reduction in the TB incidence (adjusted rate ratio [aRR] 0.59, 95% confidence interval [95%CI]:0.58–0.60), decreased TB mortality (aRR:0.69, 95%CI:0.65–0.73), and lower TB case-fatality rate (aRR:0.90, 95%CI:0.76–1.05), although not statistically significant.

In the stratified analyses according to wealth tertiles (Table 3), the effect of BFP on TB incidence showed a marked gradient and was considerably stronger among the poorest individuals (aRR:0.49, 95%CI:0.49–0.50), gradually decreasing until having only a small effect on the wealthiest individuals (aRR:0.95, 95%CI:0.93–0.98). Also, in TB mortality the BFP effect was considerably stronger among the poorest population (aRR:0.60, 95%CI:0.55–0.65), and showed a gradient.

In the stratified analyses according to ethnoracial characteristics, another gradient was evident among Indigenous, Black/pardo, and white people, both for TB incidence (aRR of 0.37 for Indigenous, 0.58 for Black/pardo, 0.67 for white), and TB mortality (aRR of 0.35 for Indigenous, 0.69 for Black/pardo, and 0.83 for white), while the gradient for TB case-fatality rate was not statistically significant (Table 3). In terms of education, the effect of BFP on TB incidence was greater in people with less education (aRR of 0.58 vs 0.65 for people with higher education) (Table 3). BFP was also associated with a higher reduction in TB mortality rates in females than in males (aRR of 0.63 vs 0.78, respectively) (Table 3).

All the sensitivity tests confirmed the robustness of the effect estimates, and the triangulation analyses showed a high degree of confidence in the impact evaluation causal inference (Appendix, p.9–15).

## Discussion

To the best of our knowledge, this is the largest and most comprehensive impact evaluation of the effects of a Conditional Cash Transfer program on an infectious disease, specifically Tuberculosis. We systematically analysed, for the first time, the program’s impact among marginalised individuals, evaluating CCT impact according to their ethnoracial and socioeconomic conditions. We observed strong and robust effects of the BFP on decreasing both TB incidence and mortality rates. Notably, BFP effectiveness exhibited a marked gradient based on ethnoracial and socioeconomic conditions, revealing significantly stronger effects among Indigenous, Black/pardo, and extremely poor populations.

Our findings exhibit a clear gradient of BFP effectiveness based on the baseline wealth level of its beneficiaries: BFP shows an extremely strong impact on TB incidence and mortality in extremely poor individuals, while demonstrating a tenfold lower effect on TB incidence in the less poor, with no discernible effects on TB mortality. This pattern is expected, as extreme poverty has been consistently shown to be a significant risk factor for the burden of TB, and TB risk levels are known to correlate with poverty levels.^21^ The BFP, through the direct transfer of money to the poorest and most marginalised families in Brazil, promotes greater access to food, both in quantity and quality, reducing food insecurity and malnutrition, an important TB risk factor, besides improving immune host defences.^6^ Moreover, housing conditions could improve, reducing crowding and poor ventilation, which are also recognized risk factors for TB.^15^ Households could also transition from cooking with burning fuels such as wood, charcoal, coal, and kerosene to cleaner fuels. This change can help reduce indoor air pollution, which has been recognized as a factor contributing to increased TB incidence. Smoking habits and alcoholism, which are strongly associated with poverty, could also decrease among BFP beneficiaries, thereby reducing their chances of being infected with TB. The prevalence of diabetes and HIV/AIDS, which is higher in the most vulnerable individuals, could also be reduced by poverty-reduction interventions.^22^ Consequently, this reduction may lead to a decrease in the incidence and mortality from TB. Moreover, individuals affected by TB who are living in poverty are more likely to avoid seeking diagnosis at health centres or to interrupt their treatment due to direct costs, such as transportation expenses, and opportunity costs, such as the difficulty of missing a day of work due to their precarious employment and subsistence conditions. These costs could represent a barrier even if prevention, diagnosis, and treatment are available free in the Unified Health System (SUS).^4^ In addition to providing monetary transfers, the conditionalities of the BFP could also contribute to reducing TB. These conditionalities are linked to requirements such as school attendance, education, and access to health services for pregnant women and children under 5 years old, which could facilitate the recognition and early diagnosis of TB.^6^ In summary, BFP promotes a greater access to income, food, and healthcare. This may lead to a reduction in TB incidence,^13^ facilitate TB early diagnosis and treatment adherence, increase the TB cure rate,^12,21,23–25^ reduce complications and death from the disease.^11,13^

Moreover, we found that BFP has an extremely strong reduction effect on TB incidence and mortality in Indigenous populations in Brazil, which have a significantly higher risk of being infected and dying from TB.^26^ While this could be explained by the same mechanisms listed above, particularly in the context of Indigenous populations the receipt of the BFP could alleviate their extreme poverty and socioeconomic vulnerability. This could reduce food insecurity and malnutrition, which are particularly high among Indigenous populations, and lessen the significant geographic barriers that hinder access to even basic healthcare services.^27^

The greater effect of BFP on TB incidence among Black and pardo people is another important finding since more than 60% of new cases of pulmonary TB in Brazil occurred among people who self-identified as Black and pardo.^4^ This could potentially be elucidated by the identical mechanisms detailed earlier. Particularly in the Black and pardo communities in Brazil, the BFP could act on historical and structural social inequities,^28^ increasing income and improving education through its conditionalities, providing access to health services and, this way, reducing the TB burden.

TB prevention and care for economically vulnerable populations became an even greater challenge during and after the COVID-19 pandemic.^14^ Each of these socially vulnerable populations has specificities and complexities when it comes to implementing TB prevention, diagnosis, and treatment interventions.^14^ Furthermore, it is important to consider that inequalities linked to local contexts, health facilities, and social, behavioural, and cultural factors, can superimpose on the organisation of health services, influencing the care provided to these populational groups.^14^ In this sense, it becomes urgent to intensify the actions of prevention and comprehensive care aimed at people in situations of social vulnerability to TB, as well as intersectoral articulations and the inclusion of the TB on the agendas of Social Assistance and Human Rights, among others. For this purpose, an Inter-Ministerial Committee for the Elimination of Tuberculosis and Other Socially Determined Diseases was recently created in Brazil.^29^

In the current Brazilian scenario, with an increase in TB cases among socially vulnerable people, the expansion of the BFP can be a beneficial strategy for TB prevention and care.^4^ It is estimated that the global economy can lose about 1 trillion dollars between 2015 and 2030 due to TB mortality.^30^ The findings of the present study reinforce that BFP reduces TB mortality, especially among populations with greater economic and social vulnerability. Thus, the BFP can be an important ally for Brazil to return to the downward trend in TB mortality rates.

Our study has certain limitations that should be acknowledged. Firstly, despite our efforts to control for all relevant confounding variables and utilise propensity score-based models with IPTW, it is important to note that these approaches may not fully account for unobservable confounding variables. To address this concern, we incorporated the average municipal rate of the TB indicator for each outcome under study (incidence, mortality, and case-fatality rate) as independent variables in logistic and Poisson regressions. These rates were estimated among individuals from the same municipalities in the cohort. This allowed us to adjust for baseline levels of the specific TB outcome under study in the municipalities and potentially associated unobservable variables, controlling for selection biases correlating PBF implementation levels with endemic TB levels in the community. Additionally, other municipal variables were also included as independent variables. Moreover, extensive sensitivity analyses were conducted, which demonstrated that the inclusion of additional independent variables related to socioeconomic inequalities, healthcare services, and municipal infrastructure did not affect the BFP effect estimates (Appendix, p.10). Second, the 100 Million Brazilians Cohort – and our derived study cohort - consists of individuals obtained from the linkage between the Unified Registry for Social Programs (CADU) and health data. It is important to note that the data included in our cohort are limited to individuals registered in the CADU, representing the poorest half of the Brazilian population. While our cohort does not include high-income individuals, it does encompass lower middle-income individuals who need to be registered in the CADU to access various governmental assistance programs. However, the external validity of the cohort at the national level is limited. Moreover, although SINAN has high sensitivity in Brazil, there may be underreporting of TB notifications.^31^ However, our study design and analytic strategy limits the possibility that underreporting could bias our results. Moreover, results from municipalities selected for high quality of vital information and low undernotifications, confirm our findings (Appendix, p. 9).

Our study presents remarkable strengths that contribute to its overall value. Firstly, we leveraged an exceptionally large longitudinal dataset, which, when combined with robust quasi-experimental impact evaluation methods, allowed us to assess the effects of interventions on an unprecedented scale. This unique approach enabled us to include a significant number of individuals and subpopulations that are often overlooked or underrepresented in traditional epidemiological studies and randomised controlled trials. This comprehensive inclusion is particularly crucial in policy evaluation, as it highlights the potential differential impacts of public interventions based on the characteristics and baseline conditions of the beneficiaries. Additionally, our study’s strength lies in the extensive range of sensitivity analyses conducted. These analyses provided further validation and reinforcement of the study’s findings, ensuring their robustness and reliability. Furthermore, employing various triangulation analyses instilled a high level of confidence in the causal inference of the impact evaluation, further bolstering the credibility of our findings.

## Conclusions

We conclude that Conditional Cash Transfer programs can greatly reduce the incidence and mortality from Tuberculosis, particularly among extremely poor, Indigenous, and Black individuals, who are usually at higher risk for TB and its devastating impacts. Therefore, the expansion of CCT programs in Low and Middle-Income Countries can significantly strengthen the global response to TB, reducing social inequalities in the TB burden, and contributing to the achievement of the End TB Strategy and the TB-related Sustainable Development Goals.

## Figures and Tables

**Table 1.** Descriptive analyses of *Programa Bolsa Famflia* (PBF) beneficiaries (BF) and non-beneficiaries (N-BF) (n =54,571,434) Brazil, 2004–2015.

	N-BF (n=30,663,476)		BF (n= 23,907,958)			
	Rates (95% CI)	No. (%)	Rates (CI)	No.	Total	
Incidence Rate	81.37 (80.97–82.13)	75,956 (47,54)	49.44 (47.84–48.50)	83,821 (52,45)	159,777	
Mortality Rate	4.68 (4.54–4.81)	4,359 (54,53)	2.08 (2.01–2.15)	3,634 (45,47)	7,993	
Case-Fatality Rate	1.37 (1.25–1.49)	495 (39,73)	0.68 (0.64–0.73)	751 (60,27)	1,246	
Social and Demographic Variables	N-BF (n =30,663,476)		BF (n= 23,907,958)		P-value^a^	SMD^b^
	No. or Mean	(%) or CI	No. or Mean	(%) or CI		
**Sex**					<0.001	0.0442
Male	14,600,007	52.4	10,800,007	54.6		
Female	16,100,007	47.6	13,100,007	45.4		
**Age**	25.32	2.14	2,417	1,607	<0.001	0.3829
**Race or ethnicity**					<0.001	0.1655
White	11,400,007	37.1	7,242,282	30.3		
Black/Pardo ^c^	19,200,007	62.1	16,400,007	68.7		
Indigenous	82,317	0.3	229,902	1.0		
**Education**					<0.001	0.1986
Illiterate, never attended school	9,979,513	32.5	8,868,743	37.1		
Elementary school	9,524,469	31.1	7,463,621	31.2		
High school	6,030,576	19.7	5,133,865	21.5		
More than high school	5,135,159	16.7	2,444,760	10.2		
**Construction material Household**					<0.001	0.0854
Bricks/cement	24,500,007	79.7	18,200,007	76.2		
Wood, other vegetal materials	6,209,340	20.3	5,687,223	23.8		
**Number of people in the family**					<0.001	0.3215
2	10,400,007	34	5,050,112	21.1		
3 a 4	14,800,007	48.3	12,400,007	52.0		
> 5	5,425,185	17.7	6,421,973	26.9		
**Per salary expenses - % MW** ^d^					<0.001	0.2856
below median	13,500,007	43.9	13,900,007	58.0		
above median	17,200,007	56.1	10,000,007	42.0		
**Lighting**					<0.001	0.2021
Electricity	27,300,007	89.1	19,600,007	82.0		
Non-electric ^e^	3,338,418	10.9	4,291,764	18.0		
**Adequate sanitation** ^f^					<0.001	0.0795
yes	20,00007	0	14,700,007	61.3		
no	10,700,007	23.9	9,252,353	38.7		
**Garbage disposal** ^g^					<0.001	0.1044
garbage collection	24,700,007	80.7	18,300,007	76.4		
burned or buried	5,919,246	19.3	5,638,928	23.6		
**Water supply**					<0.001	0.1255
Public network	23,300,007	76.1	16,900,007	70.6		
Other ^h^	7,316,985	23.9	7,028,807	29.4		
**Incidence Cohort**	123.2	89991	146	103.1	<0.001	−0.2361
*AIDS					0.060	0.0183
yes	1,355	8.8	3,009	9.3		
no	14,080	91.2	29,331	90.7		
*Diabetes					<0.001	0.1085
yes	1,236	8.0	1,716	5.3		
no	14,199	92.0	30,624	94.7		
*TDO					<0.001	0.0601
yes	8,445	54.7	16,724	51.7		
no	6,990	45.3	15,616	48.3		
*Form TB					<0.001	0.3733
yes	13,373	86.6	28,422	87.9		
no	2,062	13.4	3,918	12.1		
**Primary Health Care** ^i^	69.32	28,081	65.08	28,997	<0.001	0.1486
**Specialized clinics per 1,000 inhabitants**	0.1251	0.13958	54.17	68,916	<0.001	0.1535
**Doctors per 1,000 inhabitants**	1181	10249	1213	0.99697	<0.001	−0.0311
**Nurses per 1,000 inhabitants**	0.5863	0.38805	0.5382	0.35509	<0.001	0.3551
**Unemployment rate (%)**	7164	49317	9.02	51688	<0.001	−0.3674
**Gini index**	52.22	70554	54.17	68916	<0.001	−0.2793
**Year of entry into the cohort**					<0.001	−0.5093
2004	2,187,496	7.1	2439522	10.2		
2005	1,328,098	4.3	1942590	8.1		
2006	5,404,958	17.6	6071662	25.4		
2007	3,418,967	11.1	2105739	8.8		
2008	2,262,636	7.4	1256407	5.3		
2009	1,374,867	4.5	2175114	9.1		
2010	2,002,525	6.5	1873502	7.8		
2011	1,654,391	5.4	1684697	7.0		
2012	3525658	11.5	1599840	6.7		
2013	2044790	6.7	1141515	4.8		
2014	3022251	9.9	883651	3.7		
2015	2436839	7.9	733719	3.1		
**Obs.:**	30,663,476		23, 907,958			

a The following were used for a comparison between the groups: (i) the two-tailed t-test for continuous variables and (ii) the Pearson's chi-squared test (χ2) for categorical variables.

b SMD - Standardized mean difference.

c Race or ethnicity: Black/pardo self-declared black or mixed race people

d Proportional to the baseline minimum wage (MW).

e Lighting: Non-electric - No meter, lamps, candles, and others.

f % of the municipal population with inadequate baseline sanitation.

g % of municipality population with baseline garbage collection

h Water supply: Other - well, spring, and others.

i PHC percentage coverage

**Table 2.** Estimates of the average effect of the Programa Bolsa Famlília (PBF) adjusted Poisson model (with robust standard errors) on Tuberculosis incidence, mortality and the case-fatality rate in Brazil, 2004–2015.

Adjusted Model	Outcomes (RR^a^ - CI^b^ 95%)		
	Incidence	Mortality	Case-Fatality
**PBF**	0.59*** (0.58–0.60)	0.69*** (0.65–0.73)	0.90 (0.76–1.05)
**Sex**			
Male	1 (base)	1 (base)	1 (base)
Female	0.58*** (0.57–0.59)	0.40*** (0.38–0.42)	0.69*** (0.59–0.79)
**Age** ^c^	1.19*** (1.19–1.20)	1.63*** (1.61–1.65)	1.44 (1.38–1.50)
**Race or ethnicity**			
White	1 (base)	1 (base)	1 (base)
Black/Pardo^d^	1.42*** (1.40–1.44)	1.70*** (1.60–1.81)	1.02 (0.86–1.21)
Indigenous	3.63 *** (3.43–3.84)	4.50*** (3.59–5.65)	1.58* (0.92–2.70)
**Education**			
Illiterate, never attended school	1 (base)	1 (base)	1 (base)
Elementary school	1.83 (1.80–1.86)	1.05*** (0.99–1.11)	0.94 (0.79–1.12)
High school	2.21*** (2.17–2.25)	0.99*** (1.93–1.06)	0.77** (0.62–0.94)
More than high school	1.74*** (1.70–1.77)	0.60*** (0.54–0.67)	0.52*** (0.37–0.73)
**Construction material Household**			
Bricks/cement	1 (base)	1 (base)	1 (base)
Wood, other vegetal materials	1.22*** (1.20–1.24)	1.21*** (1.14–1.29)	1.15 (0.98–1.37)
**Number of people in the family**			
2	1 (base)	1 (base)	1 (base)
3 a 4	0.97 (0.96–0.99)	0.83*** (0.78–0.88)	0.89** (0.89–1.28)
> 5	1.20 (1.18–1.21)	0.99 (0.92–1.06)	1.07** (0.76–1.05)
**Per salary expenses - % MW** ^e^			
below median	1 (base)	1 (base)	1 (base)
above median	0.77 (0.78–0.79)	0.71 (0.66–0.75)	0.89 (0.75–1.05)
**Lighting**			
Electricity	1 (base)	1 (base)	1 (base)
Non-electric ^f^	1.34*** (1.32–1.36)	1.54*** (1.45–1.65)	1.09 (0.91–1.31)
**Inadequate sanitation** ^g^	1.00*** (0.99–1.02)	1.08** (1.02–1.15)	0.96 (0.81–1.14)
**Garbage disposal** ^h^	0.78***(0.77–0.80)	0.77*** (0.71–0.83)	1.03** (0.82–1.30)
**Water supply** ^i^			
Public network	1 (base)	1 (base)	1 (base)
Other	0.95***(0.93–0.96)	0.99 (0.92–1.06)	0.95 (0.79–1.15)
**Incidence Cohort**	1.00*** (1.00–1.00)	1.00*** (1.00–1.00)	0.99 (0.99–1.00)
**AIDS**	-	-	1.93*** (1.39–2.68)
**Diabetes**	-	-	0.87 (0.68–1.10)
**TDO**	-	-	1.54*** (1.34–1.77)
**Form TB**	-	-	0.54 *** (0.42–0.69)
**Primary Health Care** ^j^			
Coverage	1 (base)	1 (base)	1 (base)
no coverage	0.99*** (0.99–1.00)	1.00*** (0.99–1.00)	1.00 (0.99–1.00)
**Specialized clinics per 1,000 inhabitants**	0.63*** (0.59–0.67)	0.54*** (0.39–0.73)	1.15 (0.54–2.42)
**Doctors per 1,000 inhabitants**	1.11*** (1.10–1.12)	1.19*** (1.00–1.10)	1.08 (0.94–1.25)
**Nurses per 1,000 inhabitants**	0.95*** (0.92–0.98)	0.80*** (0.70–0.91)	1.11 (0.75–1.66)
**Unemployment rate (%)**	1.01*** (1.01–1.02)	1.03*** (1.02–1.04)	1.00 (0.98–1.02)
**Gini index**	0.99*** (0.99–1.00)	1.00*** (0.99–1.00)	0.98* (0.97–1.00)
**Year of entry into the cohort**	yes	yes	yes
**Obs.:**	54,565,735	54,571,434	46,344

a The following were used for a comparison between the groups: (i) the two-tailed t-test for continuous. variables and (ii) the Pearson’s chi-squared test (χ2) for categorical variables.

b SMD - Standardized mean difference.

c age categorized every 10 years.

d Race or ethnicity: Black/pardo self-declared black or mixed race people.

e Proportional to the baseline minimum wage (MW).

f Lighting: Non-electric - No meter, lamps, candles, and others.

g % of the municipal population with inadequate baseline sanitation.

h % of municipality population with baseline garbage collection.

i Water supply: Other - well, spring, and others.

j PHC percentage coverage.

**Table 3.** Estimates of the average effect of the *Programa Bolsa Famlília* (PBF), in adjusted Poisson models (with robust standard errors), on Tuberculosis incidence, mortality, and the case-fatality rate in Brazil, 2004–2015.

Adjusted Models	Incidence		Mortality		Case-Fatality	
	IRR^a^	95% CI^b^	IRR^a^	95% CI^b^	IRR^a^	95% CI^b^
**Wealth** ^c^						
**Lower wealth** ^d^	0.49***	(0.49–0.50)	0.60***	(0.55–0.65)	0.80	(0.64–1.01)
Obs.	18,476,834		18,479,518		20,752	
**Medium wealth** ^d^	0.55***	(0.54–0.57)	0.69***	(0.63–0.77)	1.08	(0.82–1.43)
Obs.	17,714,018		17,715,984		16,74	
**Higher wealth** ^d^	0.95***	(0.93–0.98)	1.00	(0.85–1.17)	0.92	(0.60–1.42)
Obs.	18,596,773		18,597,844		8,979	
Race or ethnicity	IRR^a^	95% CI^b^	IRR^a^	95% CI^b^	IRR^a^	95% CI^b^
**White**	0.67***	(0.66–0.69)	0.83***	(0.73–0.94)	1.26	(0.93–1.71)
Obs.	18,612,330		18,613,587		12,288	
**Black/Pardo** ^e^	0.58***	(0.57–0.59)	0.69***	(0.64–0.73)	0.84	(0.69–1.00)
Obs.	35,641,321		35,645,701		33,328	
**Indigenous**	0.37***	(0.32–0.42)	0.35***	(0.20–0.62)	0.16	(0.0073.33)
Obs.	312,084		312,146		717	
Education	IRR^a^	95% CI^b^	IRR^a^	95% CI^b^	IRR^a^	95% CI^b^
**Illiterate, never attended school**	0.58***	(0.57–0.59)	0.72***	(0.68–0.78)	0.90	(0.66–1.23)
Obs.	35,830,269		35,833,532		7,741	
**Elementary school**	0.58***	(0.57–0.60)	0.63***	(0.56–0.71)	0.93	(0.71–1.21)
Obs.	11,160,783		11,162,535		17,185	
**High school and University education**	0.80***	(0.77–0.83)	0.90	(0.71–1.14)	1.06	(0.84–1.35)
Obs.	7,577,703		7,578,387		25,267	
Sex	IRR^a^	95% CI^b^	IRR^a^	95% CI^b^	IRR^a^	95% CI^b^
**Male**	0.60***	0.59–0.61	0.78***	0.72–0.84	0.94	0.78–1.13
Obs.	25,432,661		25,435,955		25,681	
**Female**	0.61***	0.59–0.62	0.63***	0.57–0.69	0.90	0.66–1.23
Obs.	29,142,346		29,144,751		20,663	

Notes: All models were adjusted for the same demographic and socioeconomic variables in Table 2.

a) The following were used for a comparison between the groups: (i) the two-tailed t-test for continuous. variables and (ii) the Pearson’s chi-squared test (χ2) for categorical variables.

b) SMD - Standardized mean difference.

c) Measured by capita expenses proportional to the baseline minimum wage (MW). d Tercile 1,2 and 3 33,33% each one.

d) Lower wealth: tercile 1. Medium wealth: tercile 2. Higher wealth: tercile 3.

e) Race or ethnicity: Black/pardo self-declared black or mixed race people.

## Data Availability

The data underlying this article will be shared on reasonable request to ISC/UFBA and CIDACS/Fiocruz and after ethical approval. All data supporting the findings presented were obtained from the *Centro de Integração de Dados e Conhecimentos para Saúde* (CIDACS). Importantly, restrictions apply to access to the data, which contains sensitive information, were licensed for exclusive use in the current study and, due to privacy regulations from the Brazilian Ethics Committee are not openly available. Upon reasonable request and with express permission from CIDACS (mail to cidacs.curadoria@fiocruz.br) and approval from an ethical committee, controlled access to the data is possible. The dataset is registered under the following DOI handle: https://hdl.handle.net/20.500.12196/CIDACS/65, which provides metadata and a register of all versions of the database.

## Research in Context

To investigate the available evidence on the effect of conditional cash transfers (CCT) and tuberculosis (TB), we initially searched PubMed for studies published containing the following terms: cash transfers (CT) [MeSH Terms] OR conditional cash transfer [MeSH Terms] AND tuberculosis [MeSH Terms]. Searches were not restricted by language or date; the last search was done on 19^th^ March 2024. We also checked the referenced studies of the selected articles.

## Evidence before this study

The review identified some observational studies that explored the effect between CT programs and TB outcomes. The findings suggest that CT and CCT programs have the potential to positively influence TB prevention, diagnosis, and treatment. Cash transfers incentivize behavior change by providing financial support to individuals or households who adhere to specific health-related conditions, such as attending TB screening, completing treatment, or improving healthcare-seeking behaviour. Studies in low- and middle-income countries (LMICs) such as Ecuador, Argentina, Mexico, Brazil, Peru, India, Nigeria, Zimbabwe, Nepal, and Indonesia showed that CT programs were associated with improved TB outcomes, including increased treatment adherence and completion rates. Other observational studies indicate that CT effectively alleviates the economic burdens faced by TB-affected households. These costs, which often result from medical expenses, loss of income, and other related factors, can push affected households into poverty and hinder their access to proper TB care. Thus, these transfers help mitigate the catastrophic costs associated with TB, ensuring better access to treatment and reducing economic hardships for affected families. However, no study has ever systematically evaluated the effects of CCT programs in large cohorts of vulnerable individuals, and none has ever comprehensively estimated CCT effect according to gradients of poverty levels and ethnoracial differences, evaluating CCT impact on sequential TB burden indicators such as TB incidence, mortality, and case-fatality rate.

## Added value of this study

To our knowledge, this is the largest evaluation ever attempted of the impact of a CCT program on an infectious disease, and in particular on TB incidence and mortality. Thanks to the unprecedented dimension of its cohort of individuals, it is the first study to assess the effects of the world`s largest CCT with a unique level of granularity among socioeconomic vulnerable populations of an extremely unequal country of continental dimensions such as Brazil. We looked in particular at these effects across different ethnoracial groups, as well as levels of poverty, sex and age. Our results show that CCT programs, such as the *Bolsa Família Program*, have a strong effect on the reduction of TB incidence and mortality, especially in the Indigenous, Black, pardo and extremely poor populations.

## Implications of all the available evidence

Our results show that CCT programs, such as the *Bolsa Família Program*, have emerged as a promising strategy to improve TB-related outcomes by addressing the underlying social and economic factors that contribute to TB transmission and progression. Nationwide poverty-reduction policies could significantly reduce the burden of TB in marginalized and socially vulnerable populations, especially in LMICs.
